# Influence of Lipomannan and Lipoarabinomannan Concentration on Mycobacterial Inner Membranes Characterized by All-Atom Simulations

**DOI:** 10.1021/acs.jcim.6c01169

**Published:** 2026-07-20

**Authors:** Hwayoung Lee, Nathaniel Rygh, Matthieu Chavent, Wonpil Im

**Affiliations:** † Department of Biological Sciences, 1687 Lehigh University, Bethlehem, Pennsylvania 18015, United States; ‡ Department of Bioengineering, Lehigh University, Bethlehem, Pennsylvania 18015, United States; § Centre de Biologie Intégrative (CBI), Laboratoire de Microbiologie et Génétique Moléculaires (LMGM), 27091 Université de Toulouse, CNRS, Toulouse 31400, France

## Abstract

Mycobacteria are responsible for causing severe illnesses like tuberculosis and leprosy in humans. Studying the mycobacteria cell envelope presents a significant challenge due to its intricate lipid compositions and structural variations and also its harmful nature in a typical experiment setting. In this study, we use all-atom molecular dynamics simulations to study mycobacterial inner membranes (MIMs). By incorporating different types of phosphatidyl-*myo*-inositol-mannosides (PIMs) and their glycoconjugates such as lipomannan (LM) and lipoarabinomannan (LAM) lipoglycans, we have constructed both symmetric and asymmetric membrane systems to study the MIM structure and dynamics under varying compositions of each lipid type. Our results show that the phospholipid/PIM-rich inner leaflet remains stable and fluid, while the outer leaflet structure and dynamics are heavily governed by lipoglycan surface density. Importantly, as LM/LAM concentration increases, the polysaccharide chains shift from flexible, membrane-lying orientations to a compact brush-like state aligned with the membrane normal. This crowding significantly reduces the solvent-accessible volume and limits direct interactions between LM/LAM sugars and the outer leaflet surface. Furthermore, we observe that high lipoglycan presence in the outer leaflet slows lipid diffusion across the entire bilayer, demonstrating a dynamic coupling between the two leaflets. By resolving these LM/LAM sugar-level dynamics and their impact on membrane-wide properties, this study provides a molecular framework for future MIM modeling and simulation with various (peripheral) membrane proteins to better understand how the MIM functions as a regulated physical barrier and a platform for mycobacterial virulence.

## Graphical abstract

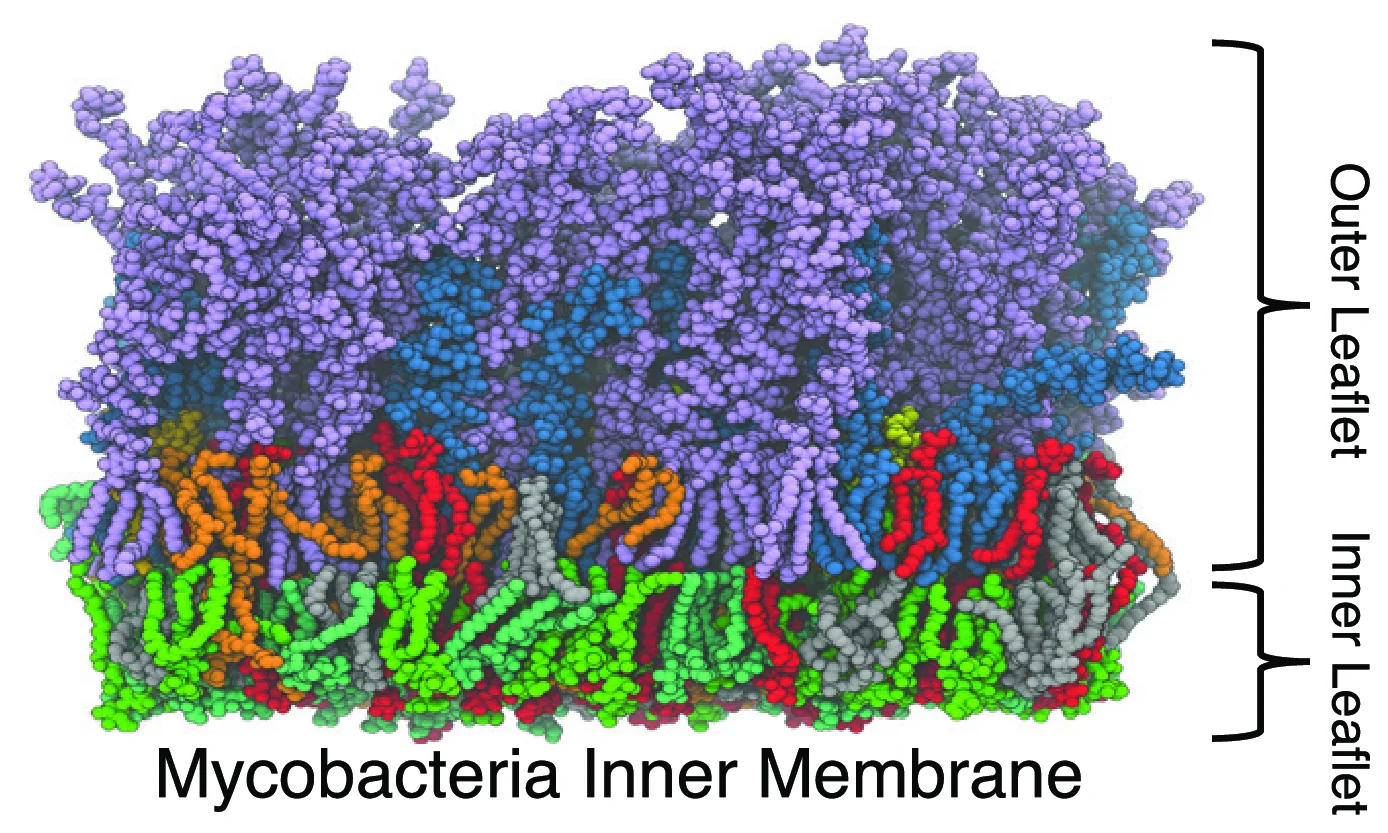

## Introduction

The complex mycobacterial cell envelope is the defining feature of the genus and a major determinant of its physiology, pathogenicity, and intrinsic resistance to antibiotics. Rather than conforming to classical Gram-positive or Gram-negative paradigms, mycobacteria possess a highly specialized, multilayered envelope composed of an inner membrane, a thick and chemically diverse peptidoglycan-arabinogalactan layer, and an outer membrane enriched in mycolic acids.^1–4^ This unusual architecture creates a formidable permeability barrier^5–7^ while simultaneously supporting a wide range of essential cellular processes. Despite its central importance, many aspects of the molecular organization and physical behavior of the mycobacterial inner membrane (MIM) remain poorly understood and have only recently begun to be explored using modern biophysical and computational approaches.^8–11^

A prominent characteristic of the MIM is the presence of complex glycoconjugates, among which lipomannan (LM) and lipoarabinomannan (LAM) lipoglycans are particularly abundant and biologically significant (Figure 1).^12–15^ Both molecules are anchored to the MIM through a phosphatidyl-*myo*-inositol-mannoside (PIM) moiety, while their extended polysaccharide domains project away from the membrane surface and into the periplasm. LM and LAM are involved in numerous aspects of mycobacterial biology, including modulation of membrane properties,^16^ interactions with host immune cells,^14,17,18^ and regulation of host–pathogen signaling pathways. LAM is well established as a key virulence factor capable of altering macrophage activation and phagosomal maturation.^13,19,20^ Beyond their immunological roles, LM and LAM are integral components of the MIM itself, yet their contributions to the MIM structure and dynamics are not well characterized.

**Figure 1:**
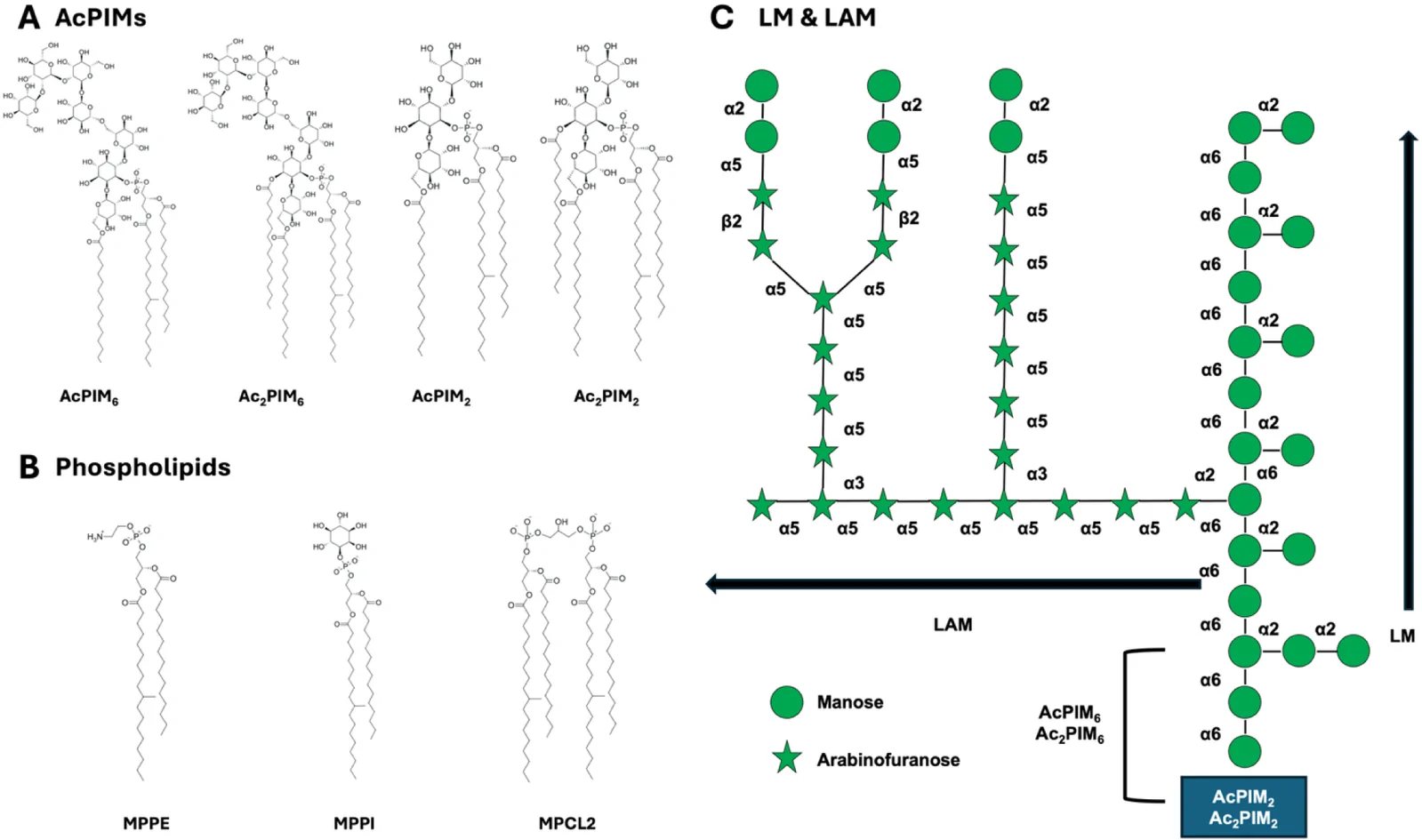
Chemical and symbolic structures of MIM lipids used in this study. (A) Chemical structures of AcPIM_2_, Ac_2_PIM_2_, AcPIM_6_, and Ac_2_PIM_6_ glycolipids, illustrating variations in glycosylation and acyl chain composition. (B) Chemical structures of phosphatidylethanolamine (PE), phosphatidylinositol (PI), and cardiolipin (CL) phospholipids: 10-methyl octadecanoyl (18:0 10-methyl) palmitoyl (16:0) PE (MPPE), MPPI, and MPCL2 with a net charge of –2e. (C) LM/LAM lipoglycan carbohydrate sequences. The directional arrows indicate the hierarchical domain organization and biosynthetic relationship between the two molecules. The arrow labeled “LM” delineates the shared structural core, consisting of the phosphatidylinositol anchor and the conserved mannan core domain. The secondary arrow highlights the additional arabinan domain and specific capping motifs that are unique to LAM.

Several unresolved questions limit our understanding of how LM and LAM function within the MIM. The relative abundance of LM and LAM in the MIM is not precisely known and may vary with species, growth conditions, or environmental stress.^14,21^ Moreover, the MIM is a fluid and compositionally heterogeneous environment with enriched PIMs that constitute a substantial fraction of total membrane lipids. Multiacylated PIM species such as Ac_2_PIM_2_, which contains four fatty acyl chains (Figure 1), are thought to promote high packing density and reduced membrane fluidity,^8,22–24^ potentially contributing to intrinsic drug resistance by limiting antibiotic influx. Within this crowded and heterogeneous lipid environment, it remains unclear how large, flexible lipoglycans such as LM and LAM behave and how their polysaccharide domains interact with surrounding lipids and with each other. Furthermore, the extent to which LAM reaches above the MIM and its interaction with the peptidoglycan and the outer membrane is unresolved. While experimental studies have provided valuable information on chemical composition and overall architecture, they offer limited access to molecular-scale structure and dynamics. For example, recent work has revealed pronounced lateral heterogeneity within mycobacterial membranes, including differences in fluidity between cell poles and sidewalls, and disruption of these patterns in LAM-deficient mutants.^24,25^ As experimental approaches generally provide ensemble-averaged or static views, it is difficult to resolve molecular-level features such as conformational flexibility of polysaccharide chains, time-dependent clustering, and coupling between glycoconjugates and the underlying lipid leaflets.

Molecular dynamics (MD) simulation provides a means to address these challenges by enabling direct observation of membrane system’s structure and dynamics at the molecular level. The computational study of complex bacterial membranes has been transformed by advances in both all-atom and coarse-grained (CG) MD simulations.^8,26–30^ Significant efforts have been devoted to the Gram-negative outer membrane,^31–35^ where the development of specialized tools such as the CHARMM-GUI^27,36–38^ LPS Modeler and Membrane Builder has enabled the systematic construction of chemically realistic membrane models. More recent simulations^8,22,39^ have expanded this framework to include diverse glycolipid chemistries and their interactions with membrane proteins, revealing how variations in sugar headgroups and acylation patterns dictate membrane organization and dynamics.

Extending these computational approaches to the mycobacterial cell envelope presents unique challenges due to the exceptional size, flexibility, and branching complexity of LM and LAM. While the mycobacterial outer membrane (MOM) has begun to receive increased attention through recent all-atom and CG simulation studies,^2,39,40^ which provide a transformative molecular architecture of the MOM, the MIM, in its full complexity, remains comparatively less explored in simulation at full compositional and atomistic detail. Recent biophysical studies have underscored the importance of lipid domains, PIM enrichment, and membrane heterogeneity in regulating MIM fluidity and host–cell interactions,^24,41^ emphasizing that LM and LAM are not merely passive membrane anchors but active regulators of the periplasmic environment. Furthermore, recent computational perspectives have highlighted the necessity of moving from individual lipid models toward complex, multicomponent assemblies to accurately capture these emergent properties.^2^ Despite these advances, a high-resolution, time-dependent description of how varying LM and LAM ratios influence the physical state of the MIM is still lacking.

In this study, we address these gaps by using all-atom MD simulations to examine various MIM model systems containing different LM/LAM, phospholipid, and PIM ratios to capture both membrane-level properties and sugar-level structural and dynamic behavior. By explicitly incorporating these large, flexible lipoglycans into a realistic environment of phospholipids and PIMs, our approach allows us to capture both global membrane organization and stability and local molecular-scale dynamics that are difficult to access experimentally. An experimental information for LM and LAM concentrations within the native MIM is currently lacking.^2^ Therefore, rather than attempting to replicate a single definitive native state, we explore a broad spectrum of surface LM and LAM densities to establish both a physiologically plausible baseline and a series of controlled physical limiting cases. This framework allows us to systematically map the concentration-dependent consequences of macromolecular crowding on MIM behavior. By characterizing how these glycoconjugates influence membrane structure and exhibit dynamic behavior within a realistic lipid environment, this study seeks to clarify their roles in mycobacterial membrane organization and to provide a framework for interpreting experimental observations and future MIM modeling and simulation with various (peripheral) membrane proteins.

## Methods

### System Building and Simulation

In this work, we have employed a stepwise modeling strategy to build complex asymmetric MIM systems. Initial simulations focus on symmetric membrane systems of both the MIM inner and outer leaflets. These systems allow us to establish baseline behavior and assess the stability of each inner leaflet symmetric and outer leaflet symmetric model system. We have then extended the simulations to asymmetric membranes using the information from both symmetric systems to better reflect a realistic asymmetric MIM. Comparison between symmetric and asymmetric systems enables us to isolate the effects of leaflet asymmetry on membrane properties and the influence of LM and LAM. Table S1 summarizes the simulation system information in this study: 3 inner leaflet symmetric systems, 6 outer leaflet symmetric systems, and 9 asymmetric MIM systems. Figure 1 shows all lipids used in this study: MPPE, MPPI, MPCL2, AcPIM_2_, and Ac_2_PIM_2_ in the inner leaflet and MPPE, MPPI, MPCL2, AcPIM_2_, Ac_2_PIM_2_, AcPIM_6_, Ac_2_PIM_6_, LM, and LAM in the outer leaflet. All membrane systems were built and equilibrated using the standard CHARMM-GUI *Membrane Builder* ^27,38^ protocol. We used 3 replicas for each system.

To eliminate initial configuration bias and ensure robust sampling of the lipoglycan conformational space, all individual LM and LAM molecules per replica were generated using the randomized initialization protocols in CHARMM-GUI.^27,36–38,42^ Starting structures were subjected to independent, randomized rigid-body rotations around the membrane normal (*Z*-axis) and distinct localized backbone conformations during the packing phase. This setup protocol ensured that production trajectories commenced from a distinct and heterogeneous lipoglycan structure ensemble.

For the inner leaflet symmetric systems, both equilibration and production simulations were carried out in the NPT (constant particle number, pressure, and temperature) ensemble using OpenMM,^43^ with the CHARMM36 force field,^44^ TIP3P water,^45^ and 150 mM KCl. The temperature was maintained at 313.15 K using Langevin dynamics,^46^ and the pressure was controlled at 1 bar under semi-isotropic conditions using a Monte Carlo barostat^47^ with a coupling frequency of 5 ps^–1^. All production simulations were run for 500 ns. A 4 fs time step was used with hydrogen mass repartitioning (HMR),^48^ and trajectory frames were saved every 100 ps. van der Waals interactions were treated with a force-switching scheme between 10 and 12 Å^44^, while long-range electrostatic interactions were calculated using the particle mesh Ewald (PME) method.^49^

To construct the outer leaflet symmetric systems, initial area-per-lipid (APL) values were estimated based on previous CG simulation studies.^8^ Because the exact outer leaflet composition is not known, multiple LM/LAM ratios were tested. The outer leaflet systems are denoted as O-10:*N*, where *N* represents the relative quantity of LM/LAM molecules. To systematically bracket the physical boundaries of molecular crowding, these systems are interpreted at two levels: the low-concentration (O-10:1 and O-10:2) and high-concentration (O-10:8 and O-10:10) systems serve as controlled physical limiting cases, while the intermediate range (O-10:4 to O-10:6) is designated as a physiologically plausible baseline based on fluidic and structural properties detailed in this study.

In total, six symmetric systems containing LM/LAM were prepared (see Table S1). Simulations were started at the lowest LM/LAM concentration and then extended to higher concentrations after careful examination of equilibration behavior. During this process, the ratio of AcPIM_2_/Ac_2_PIM_2_/AcPIM_6_/Ac_2_PIM_6_ lipids was accordingly adjusted as part of the composition testing. Finally, 9 asymmetric membrane systems were constructed by matching the system membrane area (i.e., the XY) of the three equilibrated inner leaflet symmetric compositions and three representative outer leaflet compositions (see Table S1).

Due to the system size, all production simulations of the systems containing LM/LAM were performed using GROMACS^50^ to accommodate larger system sizes in the presence of LM and LAM. To ensure comparability with OpenMM-based simulations of smaller systems, all simulation parameters were kept identical across both programs, including the CHARMM36 force field,^44,51^ TIP3P water model,^45^ and 150 mM KCl ionic concentration. All carbohydrate topologies and parameters were described using the additive CHARMM36 force field. While the fixed-charge nature of this force field can slightly overstabilize hydrogen-bonding networks and introduce minor structural rigidity over long polymer spans, it is systematically optimized and thus suitable to capture the complex glycosidic branching junctions and flexible furanose pseudorotational characteristics of mycobacterial lipoglycans.

The leapfrog integrator was used with a 4 fs time step with the HMR method,^48^ and each simulation was run for 1 μs in the NPT ensemble. Electrostatic interactions were treated using the PME method^49^ with a real-space cutoff of 12 Å. van der Waals interactions were calculated using a cutoff scheme with force-switching applied between 10 and 12 Å. Neighbor lists were generated using the Verlet scheme^52^ and updated every 20 steps with a cutoff of 12 Å. The temperature was maintained at 313.15 K using the Nosé–Hoover thermostat^53,54^ with a coupling constant of 1.0 ps, with separate coupling groups defined for the membrane and solvent. Pressure was controlled semi-isotropically using the Parrinello–Rahman barostat,^55^ with a reference pressure of 1 bar, a coupling constant of 5.0 ps, and a compressibility of 4.5 × 10^–5^ bar^–1^ in both the lateral and normal directions. All bonds involving hydrogen atoms were constrained using the LINCS algorithm.^56^ Center-of-mass motion was removed every 100 steps using linear correction, applied separately to the membrane and solvent groups. All molecular visualizations and figures were generated using VMD.^57^

### Analysis

To verify that the 1 μs production trajectories provided sufficient configurational sampling despite the slow lateral dynamics of crowded lipoglycans, a time-series convergence analysis was performed. Figure S1 shows cumulative moving averages of key thermodynamic and structural observables (simulation box dimensions and membrane hydrophobic thickness) over time. These metrics systematically stabilized within the beginning of the simulation time, confirming that the systems reached a stable thermodynamic equilibrium. The remaining time of production sampling across three independent replicas was used to analyze various lipoglycan and membrane properties.

To examine the MIM organization along the membrane normal, number density profiles were calculated along the *Z*-axis. The simulation box was partitioned into discrete slices (bins) of 1 Å width. To ensure accurate alignment throughout the trajectory, the bilayer center was translated to *Z* = 0 for every frame. The density within each bin at position *Z* was calculated by counting the number of selected atoms and normalizing it by the volume of the bin. The density in each bin was averaged over the last 500 ns trajectories to produce a time-averaged profile.

Lateral diffusion of lipids was quantified by calculating the mean squared displacement (MSD) from the trajectories using custom Python scripts utilizing the MDAnalysis^58,59^ library. To account for the inherent asymmetry or potential environmental differences between the two leaflets, lipids were first categorized into each leaflet separately, based on their center-of-mass (COM) position relative to the membrane midplane. To ensure accurate MSD tracking across periodic boundary conditions, the trajectories were unwrapped, effectively removing artificial jumps as lipids crossed the simulation box boundaries. The lateral MSD was then calculated for each lipid type using their XY COM positions:

$$\mathrm{MSD}\left(\Delta t\right)=\langle {|r_{i}^{\left(x,y\right)}\left(t_{0}+\Delta t\right)-r_{i}^{\left(x,y\right)}\left(t_{0}\right)|}^{2}\rangle$$

where ${r\;}_{i}^{\left(x,y\right)}$ represents the lateral position of lipid *i* at a given time. The MSD was averaged over all lipids within the respective leaflet and over all possible time origins (*t*_0_) to improve statistical convergence. The lateral diffusion coefficient (*D*) was then determined by performing a linear regression on the MSD vs time plot. In accordance with the Einstein relation for two-dimensional diffusion, the slope of the linear regime was used:

$$D=\frac{1}{4}\underset{\Delta t⃗\infty }{\lim}\frac{\mathrm{MSD}\left(\Delta t\right)}{\Delta t}$$

To avoid artifacts at very short time scales or poor sampling at very long time scales, the fit was performed over a robust linear range with the final results reported in cm^2^/s.

It should be noted that the lateral diffusion coefficients reported herein are subject to finite-size effects and periodic hydrodynamic interactions. As detailed by Venable et al.,^60^ diffusion constants calculated directly from MD trajectories under periodic boundary conditions (PBC) underestimate the macroscopic infinite-system diffusion. However, since all simulated systems in our study share similar lateral dimensions and matching boundary conditions, the relative differences and qualitative impacts of specific lipids on lipid diffusion remain valid and systematically comparable.

To characterize the structural order and fluidity of the lipid bilayers, the deuterium order parameter (S_CD_) was calculated for the hydrocarbon tails using a custom analysis class built on the MDAnalysis framework. For each carbon atom in the acyl chains, the unit vector was defined along the bond connecting the carbon to its attached hydrogen atom. The order parameter was then calculated using the following equation:

$$S_{\mathrm{CD}}=\left|\left\langle \frac{3\;\cos^{2}\theta -1}{2}\right\rangle \right|$$

where *θ* is the angle between the C–H bond vector and the bilayer normal, and the angled brackets denote an ensemble average over all equivalent lipids and simulation frames. When multiple hydrogen atoms were bonded to a single carbon, the S_CD_ contribution was averaged over the C–H bonds. S_CD_ values typically range from 0 (indicating complete disorder/isotropy) to 0.5 (indicating perfect alignment perpendicular to the membrane normal). Statistical errors were calculated as the standard deviation of the S_CD_ across the analyzed trajectory. After the calculation, the results of each carbon tails were plotted within each carbon tail and the average of C22 (the first acyl chain carbon bonded to the carbonyl group in the *sn*-2 chain) in each tail was plotted for the comparison. For this, S_CD_ values of each tail (Figure S2) were separated, calculated, and plotted initially (Figure S3) to choose the proper carbon selection for the comparison.

To identify distinct conformational states of LAM, hierarchical clustering analysis was performed based on pairwise structural similarities. The analysis was implemented using a custom Python workflow incorporating the Bio.PDB module for structural manipulation and the SciPy library for cluster analysis. The similarity between any two conformations was quantified using the root-mean-square deviation (RMSD). Prior to each comparison, structures were optimally aligned using the Kabsch algorithm to minimize the RMSD and remove differences due to global translation and rotation. A symmetric pairwise distance matrix was constructed, where each entry represents the RMSD between two conformations. After clustering, the final overlap structures were rendered by overlapping clustered structures using VMD.

The hydrophobic thickness of the lipid bilayer was calculated using the MDAnalysis library. To consider the periodic boundary condition, the membrane COM was centered at the origin for each frame. The bilayer was partitioned into upper and lower leaflets based on the *Z*-coordinates of the lipid head−tail interface. The hydrophobic thickness was defined as the distance (*d*) between the average *Z*-position of the C22 and C32 atoms in the top and bottom leaflets. The final values were reported as an average across the analyzed trajectory.

To characterize the LAM conformations and orientations, we calculated LAM tilt angle, residue–residue distance, and lipid contacts. The tilt angle of specific LAM residue relative to the *Z*-axis was calculated using CHARMM.^61^ The tilt angle was defined by a vector connecting two reference points: the COMs of residues AMAN10 and AARB29 of LAM shown in Figure S4. An inter-residue *Z*-distance was measured through two steps. Initially, *Z*-coordinates of specific residues (either AMAN10 or AARB29) were extracted using in-house Python script. Then, the distance between two *Z*-coordinates was calculated by subtracting the two values. To quantify contacts between LAM and the membrane, the average number of terminal sugar contacts per lipid was derived from the final 300 ns. A membrane contact was defined when any of the sugar residues (AMAN37, AMAN45, and AMAN49 in Figure S4) were within 5 Å of a membrane lipid.

To further quantify LAM properties, we calculated the end-to-end distance, radius of gyration (*R_g_*), and persistence length. The end-to-end distance was calculated by finding the root-mean-square distance between the first glycan and the five terminal glycans (AMAN21, AMAN37, AMAN45, AMAN49, and AARB29 in Figure S4) over the final 300 ns of all concentrations and replicates.

$$R_{g}=\sqrt{\frac{\Sigma_{i}m_{i}r_{i}^{2}}{\Sigma_{i}m_{i}}}$$

The radius of gyration was further decomposed into the axial components:

$$R_{g,k}=\sqrt{\frac{\Sigma_{i}m_{i}\left[r_{i,k}^{2}\right]}{\Sigma_{i}m_{i}}},,k\in \left\{x,y,z\right\}$$

To calculate the persistence length of LAM, virtual atoms were placed in the center of geometry of each connected sugar between AMAN2 and AMAN49 to isolate a singular branch. These virtual atoms were then used with the MDAnalysis PersistenceLength class over the last 300 ns of each trajectory.

To determine the volume taken up by LM/LAM above the membrane, a voxel grid composed of 2 × 2 × 2 Å^3^ units was constructed. The van der Waals radius of each atom, with the addition of 1.4 Å representing the radius of water, was used to determine the fraction of occupied voxels in 2 Å slices along the *Z*-axis. To estimate the effect of LAM concentration on water permeability, the water diffusion constant (*D*) was calculated as a function of *Z*-distance from the bilayer center for the outer leaflet symmetric systems. To do so, simulations were extended by 3 ns saving atom coordinates every 10 ps. After centering the membrane along the *Z*-axis, 260 bins of 1 Å width were constructed to encompass 130 Å on each side of the membrane. At each frame, the *Z*-position of every water molecule and their respective bin were found and the *Z*-axis MSD was calculated over 100 ps in 10 ps increments. The average MSD of all water molecules in a bin was then used to derive *D* using the slope of the bin-averaged MSD vs time via the Einstein relation (MSD = 2*D*t). This analysis was performed over all concentrations for the full 3 ns of the trajectories.

Given the continuous nature of MD trajectories, properties calculated over consecutive simulation frames exhibit significant temporal autocorrelation. We evaluate the system by tracking robust, reproducible macroscopic trends that consistently emerge across entirely independent production ensembles. Text descriptions throughout the results have been carefully framed to focus on these clear physical shifts and qualitative consistency across replicas, ensuring a rigorous interpretation of the thermodynamic ensembles without implying uncalibrated statistical certainty.

## Results and Discussion

### Mycobacterial Inner Leaflet Symmetric Membranes are Fluid

To define baseline membrane behavior, we first examined symmetric bilayer models of the MIM inner leaflet. The bilayers consisted of MPPE, MPPI, MPCL2, AcPIM_2_, and Ac_2_PIM_2_, allowing us to focus on the glycolipid composition expected to dominate the inner leaflet.^8,23,62,63^ We varied the relative amount of AcPIM_2_ and Ac_2_PIM_2_ across three compositions (I-1:1, I-1:2, and I-2:1; see Table S1 for the system name and the corresponding lipid composition) to examine how changes in AcPIM_2_ and Ac_2_PIM_2_ contents affect lipid packing and dynamics in the absence of larger glycoconjugates. Thus, these symmetric bilayers establish a simple reference system that can be used to evaluate additional effects of LM/LAM and membrane asymmetry in later simulations.

All three membrane compositions remain intact and stable bilayers with no pore formation, large-scale deformation, or lateral phase separation (Figure 2A). The density profiles along the membrane normal (i.e., the *Z*-axis) show a clear hydrophobic core in all systems with the headgroup regions at ± 22 Å and similar bilayer hydrophobic thicknesses across compositions (Figure S5). The headgroup regions are crowded with phosphates, glycerols, and sugar rings, while the lipid tails from both leaflets meet at the membrane midplane (*Z* = 0), creating a dip. The density profiles also clearly reflect the order in the number of acyl chains of each lipid type: MPCL2 and Ac_2_PIM_2_ with 4 tails > AcPIM_2_ with 3 tails > MPPE and MPPI with 2 tails (see Figure 1).

**Figure 2:**
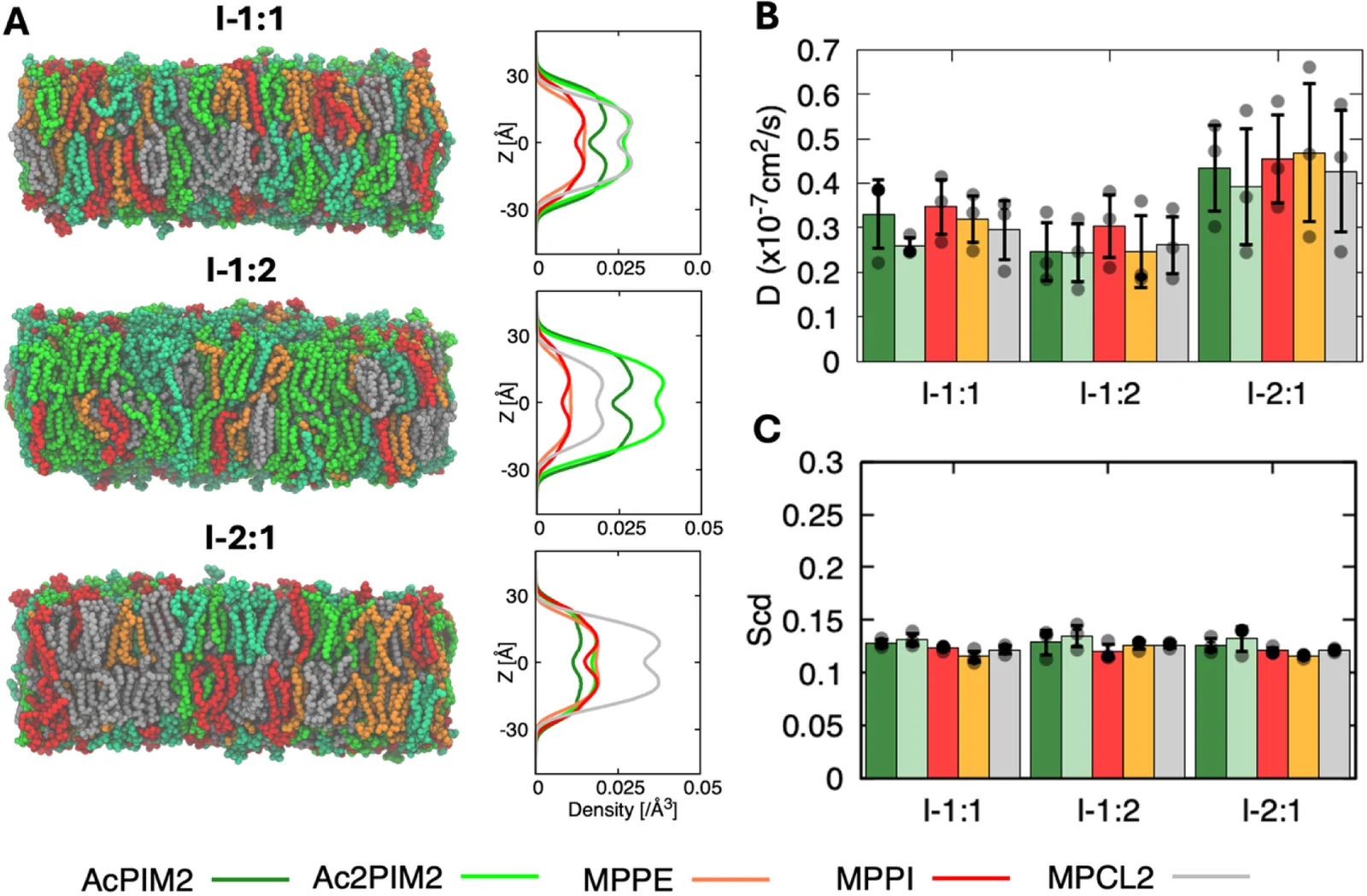
Inner leaflet symmetric membrane structure and dynamics. (A) Representative equilibrated snapshots and corresponding number density profiles along the membrane normal (i.e., the *Z*-axis) for system I-1:1, I-1:2, and I-2:1 (see Table S1 for the system name and the corresponding lipid composition) composed of phospholipids (MPPE, MPPI, MPCL2) and phosphatidylinositol mannosides (AcPIM_2_ and Ac_2_PIM_2_). (B) Lateral diffusion coefficients of individual lipid species obtained from mean squared displacement analysis. (C) Deuterium order parameters (S_CD_) of C22 carbon of phospholipid acyl chains. All systems remain structurally stable and fluid across all compositions studied. Error bars represent standard deviations among 3 replicas and individual data points are depicted as black dots over the error bars.

The lateral diffusion coefficients for all lipid species fall in the range of ∼2 × 10^–8^ to 4 × 10^–8^ cm^2^ s^–1^, consistent with lipids in fluid phase membranes (Figure 2B), which is typically reported to be on the order of 10^–8^–10^–7^ cm^2^s^–1^ in both model and biological lipid bilayers.^26,64^ While a slight increase in the mean diffusion coefficients for MPPI and MPPE is observed in the I-2:1 system, these variations remain within the calculated error margins, indicating that the overall lateral mobility remains statistically comparable across the different membrane compositions in this study. Increasing PIM content leads to a moderate decrease in diffusion, likely due to increased steric constraints at the membrane surface. Within each system, however, phospholipids and PIMs show similar mobilities, and acyl chain differences between AcPIM_2_ and Ac_2_PIM_2_ do not noticeably affect diffusion. Consistent with this, deuterium order parameters (S_CD_) are nearly identical across all composition, indicating that changes in PIM abundance do not significantly alter acyl chain packing (Figure 2C). The stability of these values suggests that the slight variations observed in lateral diffusion (Figure 2B) are likely governed by surface-level headgroup interactions or long-range lateral spacing rather than a fundamental change in the hydrophobic core’s ordering. Overall, these results show that the phospholipid-PIM membrane remains dynamically fluid and structurally stable over the compositions studied, providing a baseline for subsequent simulations including LM and LAM.

### Progressive Enrichment of LM and LAM in Outer Leaflet Symmetric Membranes Systematically Reduces Lipid Mobility

Building on the symmetric inner leaflet models, we next examined symmetric outer leaflet membranes containing the larger glycoconjugates LM and LAM (Figure 3A). As the exact lipid ratio of the MIM outer leaflet is unknown, we constructed 6 model systems with increasing LM/LAM concentration (see Table S1 for the system name and the corresponding lipid composition). All outer leaflet symmetric membrane systems remain stable over microsecond simulation time scales, but clear composition-dependent differences in structure and dynamics are observed. In each system, phospholipids (MPPE, MPPI, and MPCL2) and PIMs form a well-defined hydrophobic core with similar membrane thicknesses (Figure S5), while LM and LAM glycans occupy an extended interfacial region. As their content increases from O-10:2 to O-10:10, the density profiles along the membrane normal show a gradual broadening of the outer leaflet distribution.

**Figure 3:**
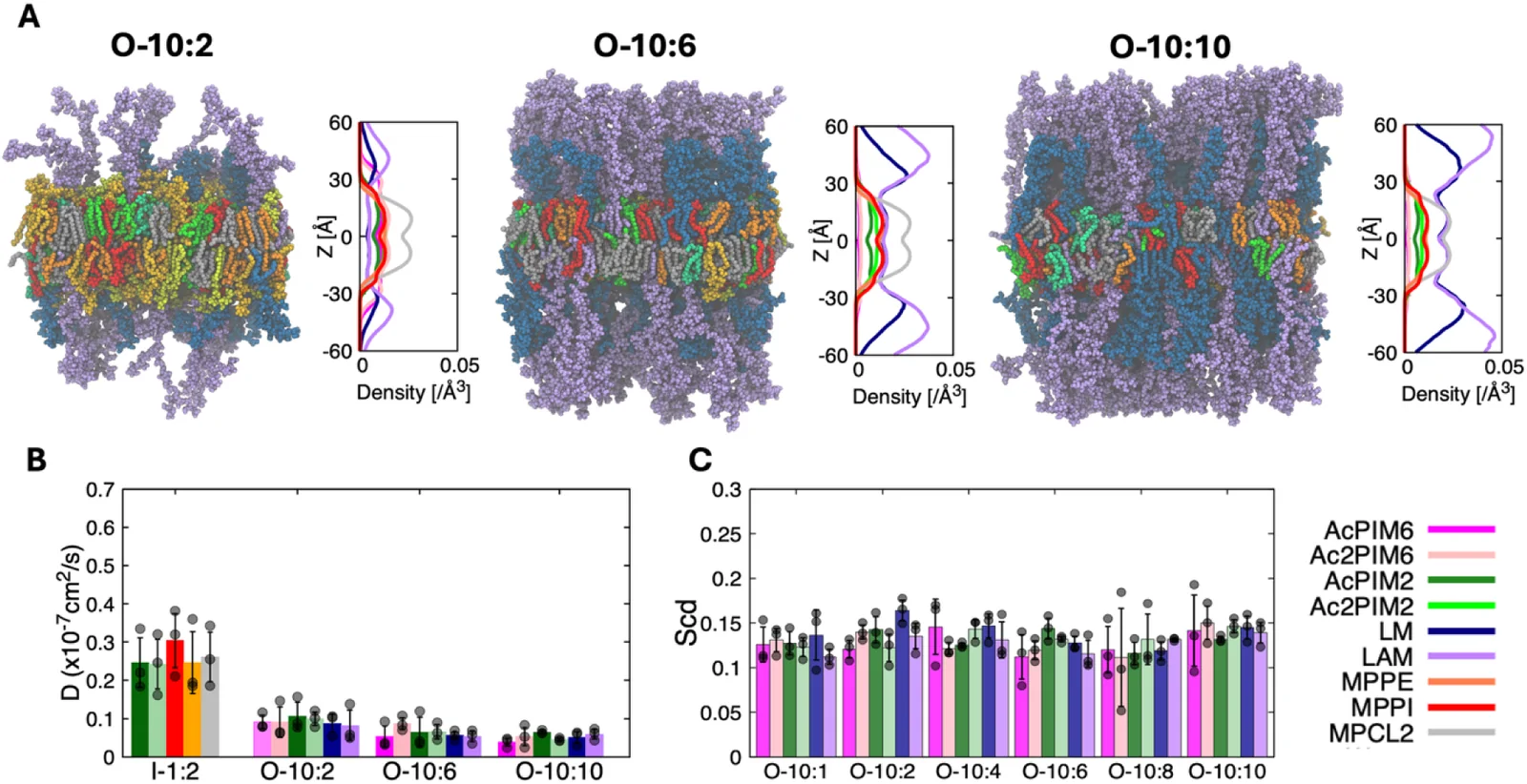
Structure and dynamics of outer leaflet symmetric membranes containing LM and LAM. (A) Representative equilibrated snapshots and number density profiles along the membrane normal for the outer leaflet symmetric membrane systems with increasing LM/LAM content (see Table S1 for the system name and the corresponding lipid composition). Each lipid is depicted with separated colors: orange for MPPE, red for MPPI, gray for MPCL2, dark green for AcPIM_2_, light green for Ac_2_PIM_2_, magenta for AcPIM_6_, yellow for Ac_2_PIM_6_, dark blue for LM, and purple for LAM. (B) Lateral diffusion coefficients of individual lipid species for the inner-leaflet reference system (I-1:2) and outer leaflet symmetric membranes (O-10:2, O-10:6, and O-10:10). (C) Deuterium order parameters (S_CD_) of C22 carbon of phospholipid acyl chains as a function of LM/LAM content. Error bars represent standard deviations among 3 replicas. The black circles represents individual data points.

LM and LAM organize into an extended polysaccharide layer that is solvated, as evidenced by the significant exposure of carbohydrate above the membrane hydrophobic area (Figure 3A). While the total bilayer density profiles highlight global changes in envelope thickness, a complete molecular breakdown showing the individual spatial distributions of the lipids along the membrane normal is provided in Figures S6 and S7. Both density analysis and visual inspection indicate that LM glycan chains adopt relatively compact but flexible conformations, whereas LAM forms larger and more diffuse structures due to its longer, branched arabinan domains. In systems with higher LM/LAM content (O-10:6 and O-10:10), neighboring carbohydrate chains interact with each other substantially, giving rise to a crowded, brush-like layer above the membrane surface. Despite this pronounced surface restructuring, the lipid acyl chains remain well packed, indicating that incorporation of LM and LAM does not destabilize the bilayer core. Instead, their primary effects are confined to the membrane surface and periplasmic space.

To evaluate how lipoglycan organization impacts water permeability along the membrane normal, we calculated the local water diffusion coefficient as a function of *Z*-distance from the bilayer center (Figure S8). At the immediate membrane interface (20 Å < *Z* < 50 Å), we observe a clear, concentration-dependent suppression of water mobility. In the densest system (O-10:10), local water diffusion drops to ∼100 Å^2^/ns whereas lower concentration systems maintain a higher rate between ∼200 and 400 Å^2^/ns. This significant reduction in localized water diffusion provides direct dynamic evidence that lateral crowding of lipoglycans near the leaflet surface forms a high-resistance physical barrier and hinders solvent permeation. Additionally, the water diffusion converges among all systems near 120 Å above the membrane center to ∼725 Å^2^/ns representing the diffusion in the bulk solvent. Collectively, these transport dynamics demonstrate that increasing lipoglycan density establishes a highly localized, potent permeation gate directly adjacent to the cell wall interface.

Lipid mobility in the outer leaflet is strongly dependent on both the concentration and identity of the glycoconjugates. As shown in Figure 3B, the lateral diffusion coefficients decrease asymptotically with increasing LM/LAM content for all lipid species, including phospholipids and AcPIMs. The reduction in diffusion is most pronounced in LAM-rich systems, consistent with the larger size and higher branching of LAM relative to LM. These trends suggest that glycoconjugates restrict lateral motion through steric crowding between glycan chains, resulting in a less dynamic outer leaflet at high glycoconjugate loadings. On the other hand, lipid packing shows a very similar result, regardless of the LM/LAM content (Figures 2C and 3C). Together, these results point to the development of lateral heterogeneity with glycoconjugate-enriched regions exhibiting higher rigidity and reduced dynamics. Overall, our findings indicate that LM and LAM concentration serves as a primary biophysical determinant of outer leaflet structure and dynamics, producing a thick, hydrated surface layer. This architectural remodeling provides a plausible structural framework that may influence membrane permeability, mechanical resilience, and the spatial accessibility of surface epitopes involved in host–pathogen interactions.

### Molecular Crowding Reshapes the Conformational and Orientational Landscape of LAM

The metrics discussed so far establish the general stability of both MIM inner leaflet and outer leaflet symmetric membrane systems. We expect that the LAM surface density does not just alter global properties, but it restricts the polysaccharide domains movement, orientation, and the solvent-accessible volume in the periplasmic space due to physical crowding. Figure 4A shows representative snapshots and structural overlays of LAM from the low and high concentration systems.

**Figure 4:**
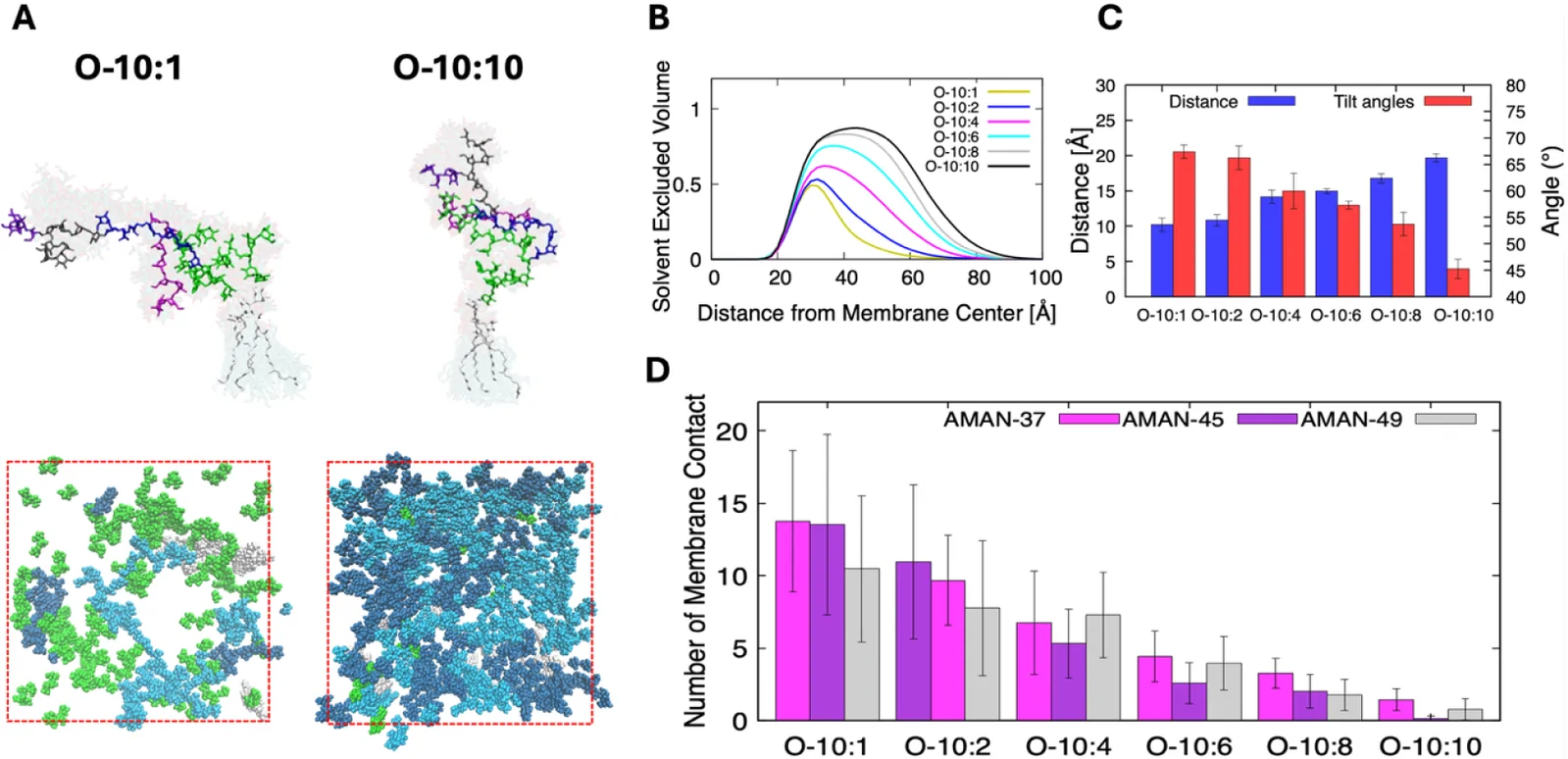
Concentration-dependent structure and dynamics of LAM in a mycobacterial inner membrane. (A) Representative snapshots and overlaid conformations of LAM extracted from low and high LAM concentration systems. Red dashed boxes indicate the primary simulation system XY area. (B) Solvent excluded, LM/LAM-occupied volume profiles as a function of distance from the membrane center (*Z* = 0) for systems O-10:1, O-10:2, O-10:4, O-10:6, O-10:8, and O-10:10. A value of 1 represents that all volume is occupied by LM/LAM and thus inaccessible to solvent. (C) Average *Z*-distance between LAM sugar residues AMAN10 and AARB29 in Figure S4 (blue bars, left axis) and average tilt angle of a vector from AMAN10 to AARB29 relative to the membrane normal (red bars, right axis) for systems O-10:1 to O-10:10. (D) Average number of contacts formed between LAM sugar residues (defined in Figure S4) and the membrane lipids for systems O-10:1 to O-10:10. Error bars represent standard deviations among 3 replicas.

At low surface densities (O-10:1), LAM molecules exhibit high flexibility, extending laterally parallel to the membrane surface while the sugar residues sample a wide range of orientations. As the concentration increases toward the O-10:10 regime, however, these architectures become significantly more compact, lose substantial flexibility, and align vertically along the membrane normal. Structural overlays from this analysis indicate that higher LAM surface density physically limits the conformational freedom of the polysaccharide chains, a clear signature of steric crowding driven by localized lipoglycan-lipoglycan interactions (Figure 4A).

Conformational clustering of these trajectories yielded two primary, highly populated global ensembles, reflecting these stable and converged configurations of the core glycan domains during the production time scale. These geometric and ensemble properties provide a structural basis for the concentration-dependent assembly of the outer mycobacterial membrane matrix.

As LAM concentration increases, steric crowding above the membrane forces the glycan chains to become more vertical and reach further into the periplasmic space. This elongation is visible in the average *Z*-distance between the sugar residues AMAN10 and AARB29 (Figure 4C). As we move from O-10:1 to O-10:10, the mean *Z*-distance increases, demonstrating that the terminal sugars are moving farther from the membrane. This is simultaneously accompanied by a decrease in the average tilt angle of a vector from AMAN10 to AARB29 (Figure 4C). Essentially, as LAM concentration increases, the polysaccharide chains reorient themselves to lie more perpendicular to the membrane surface. This transition to a more vertical conformation at higher concentrations is substantiated by the increasing *Z*-axis radius of gyration and end-to-end distances (Figures S9 and S10). Additionally, the increasing rigidity is reflected in higher LAM persistence lengths as surface density increases (Figure S11). We also calculated the solvent excluded volume (SEV), i.e., LM/LAM-occupied volume relative to the distance from the membrane midplane to quantify LM/LAM’s spatial occupancy (Figure 4B). In the low concentration systems, the SEV distributions are low, confirming that the sugar residues are largely solvent exposed, free to move, and stay primarily near the membrane-solvent interface. In the O-10:8 and O-10:10 systems, however, the SEV rises significantly, especially further away from the membrane. This agrees with the observed elongation of LAM as concentration increases. At these higher concentrations, LM/LAM takes up a significant portion of space above the membrane (∼80%), providing a significant barrier to molecular permeability, as demonstrated by the Z-dependent water diffusion profiles in Figure S8.

Finally, we examine the number of atom contacts between LAM sugars and the lipid molecules (Figure 4D). These interactions are biologically relevant given that arabinosyltransferases involved in LAM biosynthesis are membrane-associated enzymes, implying that transient contacts between LAM carbohydrate domains and the membrane surface is necessary during LAM elongation.^7,65,66^ Our analysis shows that the O-10:1 and O-10:2 systems have the highest number of contacts. This is expected as LAM at low concentration has the most conformational freedom to bend down to make membrane contact. As concentration increases, the number of contacts per molecule drops significantly. At higher LAM surface densities, neighboring LAM molecules crowd one another, reducing the available space for individual carbohydrate chains to spread across the membrane surface. Instead, the chains tend to adopt more upright conformations, which limits their ability to maintain extended contacts with lipids. Under these conditions, interactions between neighboring carbohydrate domains become more prevalent than direct carbohydrate-lipid contacts.

Taken together, these simulations indicate that LAM concentration strongly influences the conformational organization and membrane interaction behavior. At low surface densities, LAM remains flexible, highly solvent-exposed, and capable of frequent membrane contact, whereas crowding at higher densities drives a transition toward a more compact, membrane-normal orientation with reduced lipid interactions. Given that LAM abundance fluctuates with physiological state, particularly during the transition to stationary phase, our results suggest that such variations act as a structural switch for cell envelope remodeling. By modulating surface density, the bacteria can effectively control LAM orientation and its subsequent interactions with the host environment.^67^ Although LAM and LM are key components of the mycobacterial cell envelope and important virulence factors, their precise spatial organization relative to the arabinogalactan-peptidoglycan complex and whether LAM extends fully through the peptidoglycan layer in vivo warrant further investigations.^68^

### Lipid Diffusion is Primarily Dictated by Outer Leaflet LM and LAM Concentration, Demonstrating Asymmetrical Contributions of Each Leaflet

To extend the symmetric membrane analysis toward a more physiologically relevant model, we constructed asymmetric bilayers in which AcPIM_6_, Ac_2_PIM_6_, LM, and LAM are present exclusively in the outer leaflet (Figure 5). As shown in Table S1, in our 9 asymmetric MIM models, the inner leaflet is composed of phospholipids, AcPIM_2_, and Ac_2_PIM_2_ at the same ratios used in the symmetric inner leaflet models, while the outer leaflet has increasing levels of LM and LAM corresponding to O-10:2, O-10:6, and O-10:10 compositions. Given the limited experimental data on MIM lipid composition, particularly the relative ratios of glycoconjugates (AcPIMs, LM, and LAM), these systems also serve to evaluate a range of reasonable inner-outer leaflet composition combinations. This design allows direct assessment of leaflet coupling in the asymmetric MIMs. Throughout the production trajectories, the asymmetric lipid bilayers remained stable, maintaining their structural integrity and baseline dimensions across all simulated lipoglycan concentrations.

**Figure 5:**
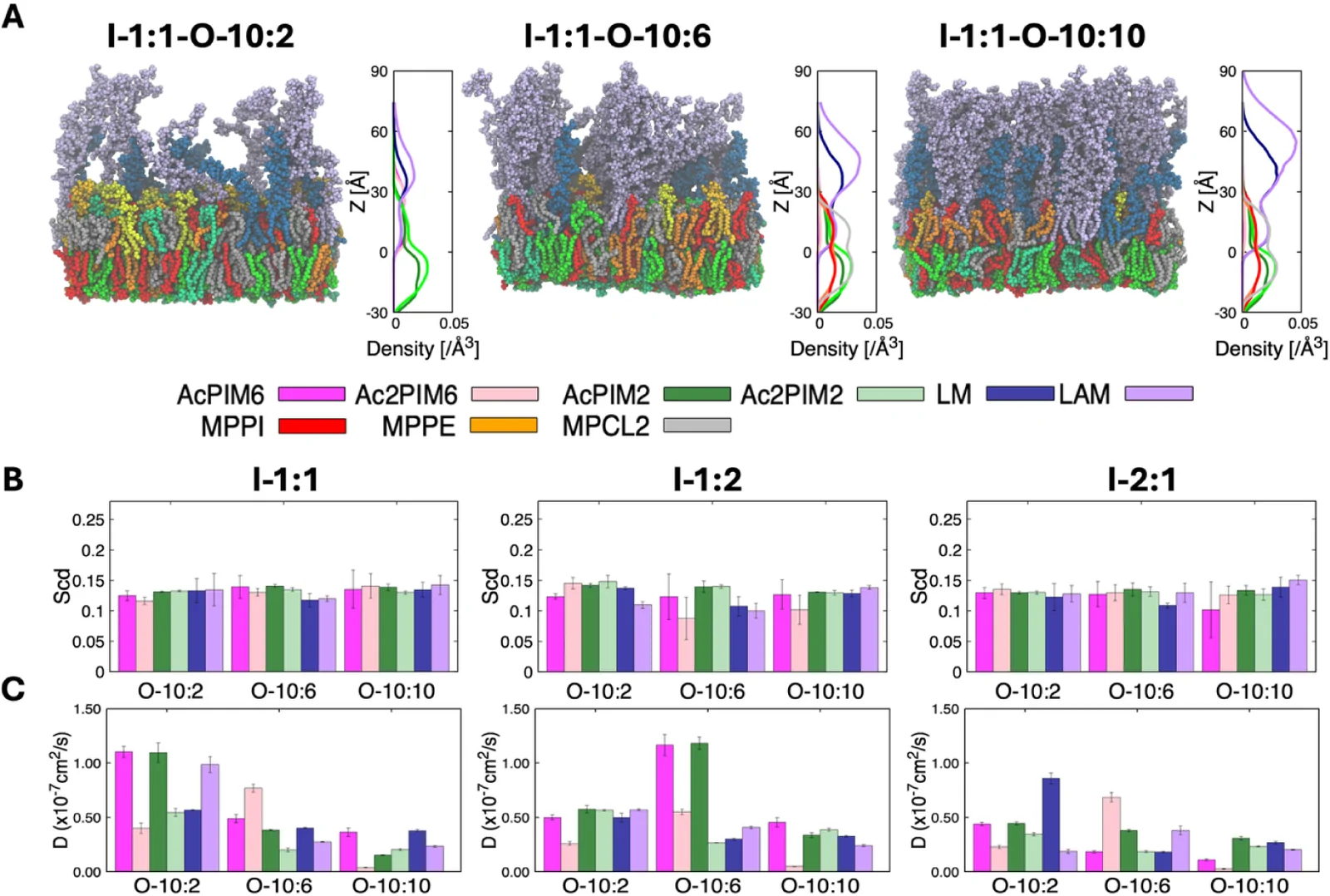
Structure and dynamics of asymmetric mycobacterial inner membrane systems. (A) Representative snapshots and number density profiles illustrating leaflet asymmetry. (B) Lipid acyl chain order parameters (S_CD_) of C22 carbon of acyl chains for outer leaflet phospholipids. (C) Lateral diffusion coefficients of lipid species in asymmetric bilayers. Increasing outer leaflet lipoglycan content reduces lipid mobility in both leaflets, demonstrating long-range dynamic coupling mediated by the lipoglycan-rich outer leaflet.

Structure analysis reveals that lipid distribution largely follows trends shown in their respective symmetric systems. The inner leaflet retains a compact hydrophobic core that closely resembles the symmetric inner leaflet systems and shows little sensitivity to changes in the opposing leaflet. The outer leaflet also demonstrates similar trends to the symmetric systems. As the LM and LAM content increases, the *Z*-dependent density profiles show a gradual extension of the outer leaflet region, consistent with the formation of an increasingly prominent, polysaccharide layer that extends into the solvent (Figure 5A). As a result, there is an increase in effective membrane thickness driven primarily by LAM extension away from the membrane surface. LM and LAM carbohydrate domains organize into an extended surface layer whose structure depends on glycoconjugate content. At lower concentrations, individual LM and LAM molecules remain relatively separated, whereas higher concentrations leads to significant contacts between neighboring carbohydrate chains. In LM/LAM-rich systems, this contact produces a dense, brush-like layer at the membrane surface. Despite this pronounced surface crowding, the inner leaflet remains well packed, indicating that bulky outer leaflet glycoconjugates do not disrupt inner leaflet organization or compromise bilayer integrity. Similar acyl chain orders are consistent with these trends (Figure 5B; see also Figures 2C and 3C). For example, outer leaflet AcPIMs stays within a similar range without noticeable differences in order with increasing LM/LAM concentration, maintaining almost identical order parameters.

Lipid dynamics reflects this leaflet-specific behavior, though the lateral diffusion coefficients (*D*) exhibit a more complex dependence on membrane composition than the structural metrics alone. Rather than a linear decrease in mobility, the asymmetric models maintain relatively similar diffusion rates between the O-10:2 and O-10:6 systems. In fact, for certain lipid species, a slight increase in *D* is observed as glycan concentration shifts from O-10:2 to O-10:6 (Figure 5C; see also Figure 3B). This suggests that at intermediate glycan densities, the membrane environment remains sufficiently fluid to accommodate additional glycans without imposing immediate kinetic restrictions. However, once the surface density is further increased to O-10:10, diffusion coefficients drop significantly across all species, marking a clear transition to a crowding-induced regime where molecular packing significantly limits lateral mobility. In contrast, lipid diffusion does not change while varying the inner leaflet composition. This demonstrates that the outer leaflet, and LM/LAM concentration, has more influence on diffusion than the inner leaflet. (Figure 5C; see also Figure 2B). This difference highlights a dynamic separation between the two leaflets where LM and LAM play the largest role in dictating lipid diffusion in the outer leaflet.

The observed lateral mobility of LM and LAM molecules presents a distinct physical profile compared to lipopolysaccharides (LPS) found in the outer membrane (OM) of Gram-negative bacteria. All-atom MD simulations of LPS-containing systems have characterized the OM outer leaflet as a nearly immobile, gel-like lattice.^69,70^ In such systems, divalent ions such as Mg^2+^ or Ca^2+^ act as an electrostatic glue between the phosphate groups of lipid A, imposing a rigid barrier that severely restricts lateral diffusion.^71,72^ This rigidity is a direct consequence of the divalent cation-mediated cross-linking between LPS phosphate groups. In contrast, the mycobacterial lipoglycans (LM and LAM) lack such strong, charge-driven intermolecular interactions, as the primary interactions between neighboring chains are mediated by transient hydrogen bonding and van der Waals forces.^62^ Consequently, LM and LAM remain significantly more mobile than LPS, allowing the mycobacterial inner membrane to retain its fluid character even under high glycoconjugate concentrations. This suggests that the mycobacterial cell envelope achieves its protective barrier properties through a thick, hydrated polysaccharide buffer that maintains a degree of fluid character, potentially facilitating the lateral reorganization of membrane proteins and cell wall biosynthetic machinery. These leaflet-specific dynamics likely serve a dual evolutionary purpose, providing a robust permeability barrier against exogenous antibiotics while preserving the membrane plasticity necessary for host–pathogen interactions and the constant remodeling of the mycobacterial cell wall.

## Conclusions

In this work, we present an all-atom MD simulation study of the MIM with a focus on the role of its major glycolipids (AcPIM_2_, Ac_2_PIM_2_, AcPIM_6_ and Ac_2_PIM_6_) and lipoglycans (LM and LAM). By systematically varying the concentration of these molecules in both symmetric and asymmetric membrane models, we explore how changes in their loading influence membrane structure, dynamics, and interfacial organization at the molecular resolution.

Our simulations provide rigorous biophysical measurements of the concentration-dependent modulation of lipid packing, lateral diffusion, glycan conformation, and local water mobility along the membrane normal. However, broader functional consequences, such as the emergence of a molecular permeability barrier or a steric shield affecting peripheral protein accessibility, are inferred implications suggested by these physical baselines. In a native mycobacterial cell envelope, these functional outcomes do not operate in isolation; rather, they are heavily affected by cell-wall proteins, osmotic gradients, and the dense outer mycomembrane layers. Isolating these baseline MIM biophysics in a reduced model system is a necessary first step to characterize the fundamental properties driving envelope behavior, and thus these functional hypotheses should be calibrated as underlying biophysical tendencies rather than definitive measures of macroscopic cellular regulation.

When evaluating the physiological relevance of the explored composition ranges, our findings strongly point to the intermediate surface densities (O-10:4 to O-10:6) as the most plausible representation of typical mycobacterial physiology. Systems within this intermediate window maintain a fluid, dynamically heterogeneous bilayer consistent with experimental observations of native membrane fluidity,^41^ while providing a balanced periplasmic glycan density. This density is sufficient to form a meaningful molecular barrier and also to preserve the conformational flexibility required for cell wall remodeling and membrane-associated enzymatic activities, such as those of arabinosyltransferases.^31–33^ Conversely, the extreme lower and upper boundaries of our composition spectrum function as physical limiting cases. They isolate the concentration dependence of lipid dynamics and glycan orientation, demonstrating how the membrane becomes rigid or hyper-crowding when driven outside typical physiological bounds.

The observation that outer-leaflet LM/LAM enrichment systematically dampens inner-leaflet lipid diffusion highlights a robust trans-leaflet dynamic coupling. In the absence of large-scale membrane undulations, this coupling is mediated primarily by local packing constraints. Steric crowding among the massive glycan trees drives a lateral condensation and acyl-chain lengthening within the outer leaflet. To maintain a cohesive, continuous hydrophobic core and minimize interfacial line tension, the inner-leaflet acyl chains are mechanically constrained to match this localized ordering. The resulting reduction in localized free volume and increased chain–chain friction across the midplane effectively couples the lateral dynamics of the two asymmetric leaflets, drawing the inner leaflet into a less fluid, more tightly packed state.

Experimental studies have long suggested that the MIM (or mycobacterial plasma membrane) differs fundamentally from canonical bacterial plasma membrane, particularly due to the abundance of PIM lipids and large lipoglycans.^23,62^ Consistent with this view, our simulations show that the inner leaflet, enriched in phospholipids and PIMs (AcPIM_2_ and Ac_2_PIM_2_), remains in a liquid-disordered state across all conditions examined in this study. Variations in PIM content primarily affect interfacial interactions and lipid organizations, with no significant changes in hydrophobic core packing or bilayer thickness.^2^ This behavior aligns with experimental observations reporting preserved membrane fluidity in mycobacteria despite substantial remodeling of lipid composition.^24^

In contrast, the properties of the outer leaflet are strongly modulated by LM and LAM concentration. Increasing lipoglycan abundance leads to reduced lateral mobility and an extension of LAM into the periplasmic space. These findings are consistent with biophysical studies suggesting that LM and LAM form an extended, hydrated layer at the membrane surface and contribute to the unusual thickness and heterogeneity of the mycobacterial envelope.^41^ Our results provide a molecular interpretation of these observations, showing how increased surface density of large polysaccharide molecules directly alters membrane dynamics and interfacial structure. At higher surface densities, LAM undergoes a gradual shift from flexible, solvent-exposed conformations to more compact and orientationally ordered states. This crowding-induced reorganization reduces the frequency of direct sugar-lipid contacts while promoting a more uniform alignment of polysaccharide chains with respect to the membrane. Such behavior is consistent with polymer brush models that have been proposed to describe cell-surface glycans and supports the idea that LAM can act as a dynamic, concentration-dependent regulator of the periplasmic-facing environment rather than as a static structural component.^73,74^

These observations reinforce the view that the MIM is an active and highly organized membrane rather than a simple permeability barrier. Previous work has suggested that glycolipid and lipoglycan composition influences antibiotic susceptibility and membrane-associated processes in mycobacteria.^16,75^ Our results provide a physical basis for these effects by showing how LAM crowding can limit access to the membrane surface and reshape the interfacial landscape. The coexistence of a fluid inner leaflet and a densely decorated outer leaflet likely enables mycobacteria to balance membrane flexibility with protection under diverse environmental conditions.

It is worth noting that the structural and dynamical trends characterized in our all-atom trajectories strongly align with the previous CG modeling of MIM outer leaflets.^2,40^ Both scales capture a consistent physical narrative. Crucially, however, our all-atom models extend these valuable CG baselines by providing the chemical resolution necessary to quantify discrete, atomistic contact frequencies with core phospholipids, and the spatial retardation of localized water molecules. Bridging these two computational resolutions validates the structural stability of these crowded assemblies while providing the atomic precision needed to understand interfacial cell-wall mechanics.

Most computational efforts to date have focused on the mycobacterial outer membrane, particularly its mycolic-acid rich architecture and low permeability. By comparison, the inner membrane has received far less attention at comparable resolution. Our results highlight an important distinction between the two membranes. Unlike the rigid outer membrane, the MIM remains fluid even at elevated lipoglycan concentrations.^24^ At the same time, both membranes rely on complex carbohydrate parts to modulate organization and dynamics, suggesting a conserved envelope-level strategy in which sugars play a central regulatory role.

Overall, this study provides a molecular framework for understanding how LM and LAM contribute to the physical properties of the MIM and complements existing experimental and modeling efforts. The models presented here should aid in interpreting observations related to membrane heterogeneity, altered glycolipid composition, and LAM-deficient phenotypes.^16,76^ Future work could extend this approach to include membrane proteins or to explore the effects of environmental variables, such as pH and ionic strength, on glycoconjugate organization. Together, these efforts will help clarify how the unique architecture of the MIM contributes to virulence and drug resistance.

## Supplementary Material



## Data Availability

The input and restart files necessary for the continuation of all simulations are freely available in a Zenodo data set (10.5281/zenodo.19239629).

## Notes

System information with lipid compositions (Table S1), lipid structural information (Figures S2 and S4), and additional analysis results (Figures S1, S3, and S5–S11) (PDF)
